# Leptin concentration and risk of coronary heart disease and stroke: A systematic review and meta-analysis

**DOI:** 10.1371/journal.pone.0166360

**Published:** 2017-03-09

**Authors:** Han Yang, Wenzhi Guo, Jie Li, Shengli Cao, Jiakai Zhang, Jie Pan, Zhihui Wang, Peihao Wen, Xiaoyi Shi, Shuijun Zhang

**Affiliations:** 1 Department of Hepatobiliary and Pancreatic Surgery, the First Affiliated Hospital of Zhengzhou University, Zhengzhou, Henan Province, China; 2 Key Laboratory of Hepatobiliary and Pancreatic Surgery & Digestive Organ Transplantation of Henan Province, Henan Province, China; University of Utah, UNITED STATES

## Abstract

**Background and purpose:**

Although high leptin concentration has been shown to be correlated with established vascular risk factors, epidemiologic studies have reported inconclusive results on the association between leptin and cardiovascular diseases (CVD). Therefore, a meta-analysis was performed to evaluate this issue.

**Methods:**

We searched Pubmed, Embase, and the Cochrane Library from their inception to Jan 2016 for both case-control and cohort studies that assessed leptin concentration and CVD risk. Reports with odds ratio (OR), risk ratio (RR) and corresponding 95% confidence intervals (CI) were considered. The data were extracted by two investigators independently.

**Results:**

A total of 13 epidemiologic studies totaling 4257 CVD patients and 26710 controls were included. A significant inverse association was shown between leptin and coronary heart disease (CHD), with an overall OR of 1.16 (95% CI: 1.02–1.32), but not for stroke (OR = 1.21, 95% CI 0.98–1.48) under sociodemographic adjustment. Further adjustment for additional cardiovascular risk factors resulted in ORs of 1.16 (95% CI 0.97–1.40) for CHD and 1.10 (95% CI 0.89–1.35) for stroke. The findings remained when analyses were restricted to high-quality studies and indicated OR estimates of 1.07 (95% CI 0.96–1.19) for CHD and 0.98 (95% CI 0.76–1.25) for stroke. In a subgroup meta-analysis, a high leptin level was not independently associated with CHD in both females (OR = 1.03, 95% CI 0.86–1.23) and males (OR = 1.09, 95% CI 0.95–1.26) or with stroke in both females (OR = 1.13, 95% CI 0.87–1.47) and males (OR = 0.80, 95% CI 0.59–1.09). There was no significant publication bias as suggested by Egger test outcomes.

**Conclusions:**

Our findings indicate that high leptin levels may not be associated with risks of CHD and stroke. Further large, well-designed prospective cohort studies are needed to fully evaluate the role of leptin on the risk of CVD.

## Introduction

Cardiovascular disease (CVD), especially coronary heart disease (CHD) and stroke, remains the leading cause of death and has become the most prominent health problem in developed and developing countries [1–4]. In recent decades, exploring risk factors for CHD and stoke and finding ways to reverse this global problem have aroused particular attention. Obesity is rapidly increasing worldwide and has been recognized as an important risk factor for CHD and stroke [5, 6]. It is hypothesized that adipokines, proteins secreted by adipocytes, possibly mediate the effects of obesity on the risk of CVD in the underlying biological mechanism [7–11].

Leptin, an adipokine hormone, plays an important role in neuroendocrine function and metabolic processes [12, 13]. Levels of leptin in humans increase with obesity and are higher in females than in males [14]. Existing studies have indicated that a potential role of leptin on CVD risk factors includes blood pressure regulation, insulin sensitivity, glucose regulation, fatty acid catabolism, platelet aggregation, angiogenesis, and inflammatory vascular responses [15–20]. However, the association between high leptin concentration and risk of CVD is controversial. A published meta-analysis, comprising eight nested case-control studies with a total of 1980 CVD patients and 11567 participants, indicated a significant association between leptin and pathogenetic risk of CHD and stroke [21]. In contrast, the latest research studies did not find the same association [22–24]. Given the inconsistency of prior results, we performed an updated meta-analysis to investigate the relationship between high leptin concentration and risk of CVD.

## Methods

### Publication search

A systematic search using electronic databases including Pubmed, Embase and the Cochrane Library with no language restrictions was performed for articles published before Jan 2016. The search terms used were (“cardiovascular diseases” OR “stroke” OR “coronary disease” OR “myocardial infarction” OR “CHD”) AND (“leptin” OR “LEP” OR “obese protein” OR “obese gene product” OR “adipokine” OR “adipocytokine”). Review articles and reference lists based upon these articles were manually obtained to identify additional pertinent studies.

### Inclusion and exclusion criteria

The following inclusion criteria were used for the study selection:(1) evaluation of leptin with risk of CHD or stroke;(2) study design using a case-control study or cohort study;(3)odds ratio (OR), risk ratio (RR) and the corresponding 95% confidence interval (CI) were reported. In addition, studies were excluded if CHD or stroke patients were included in the baseline population and all relevant reviews, reports, letters and used overlapping data were published by the same first author.

### Data extraction

Two investigators (HY and SC) individually assessed potentially relevant articles for eligibility. The following data were extracted for included studies: study characteristics (the first author’s name and year of publication), participant characteristics (country of origin, sample size, mean age or age range, and gender), leptin assessment, duration of follow-up, type of outcome (CHD or stroke), analysis strategy and results (including data to calculate its precision, such as 95% CI, standard error, or *P* values).

### Methodological quality assessment

Two reviewers (JP and JZ) independently assessed the study quality according to the scale of the Newcastle-Ottawa, including the selection of study groups, comparability of groups, and ascertainment of either the exposure or outcome of interest for case-control or cohort studies. The high-quality study was defined as a study with ≥7 awarded stars.

### Statistical analysis

The included studies reported RR for cohort studies and OR for case-control studies. These two values were assumed to be approximately equal. Heterogeneity among studies was conducted by I^2^ statistic and χ^2^ test [25]. If the value was less than 0.10 and I^2^ exceeded 50%, then we considered there to be substantial heterogeneity and a random-effect model was applied to pool the data. Otherwise, a fixed-effect model was used. Egger’s test was used to evaluate publication bias. *P<*0.05 for Egger’s tests was considered to be representative of a significant statistical publication bias.

## Results

### Literature search

**Fig 1:**
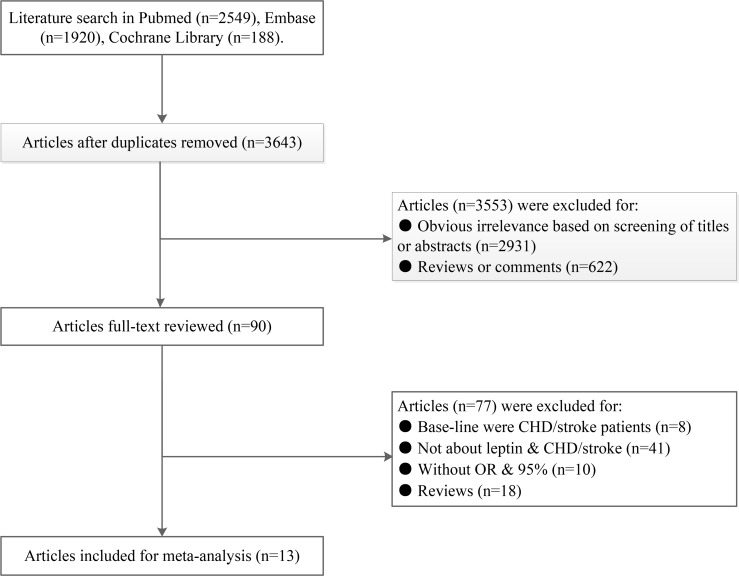
Flow of study identification, inclusion, and exclusion.

The flow diagram summarizing the process of the study search and selection is shown in Fig 1. A total of 4657 relevant studies were identified from the initial literature search, 90 of which appeared to be relevant to the meta-analysis following the subsequent selection. After careful screening and independent selection, thirteen eligible studies met all the inclusion criteria [24, 26–37], including five studies exploring the association between leptin and risk of CHD, four studies for stroke and four studies for both.

### Study characteristics

The detailed characteristics of these studies are summarized in Table 1. A total of 26710 participants were included in the final analysis, with sample sizes ranging from 140 to 6502 in individual studies. Eleven of the included studies were nested case-control studies and two were case-control studies. These studies were published between 1998 and 2015. Seven were conducted in Europe, five in the U.S.A. and one in Asia. Ages of the participants ranged from 20 years to over 90. Nearly all included studies adjusted for sociodemographics (age, race and town), and common cardiovascular risk factors (diabetes, lipids, systolic blood pressure, smoking status and body mass index).

**Table 1:** Characteristics of included studies on leptin and risk of CHD and Stroke.

First author, year	Study design	Study location	Year of baseline survey	Mean duration of follow-up (yrs)	Study population	Age Range (yrs)	Type of outcome	No. of cases
Seth S.Martin(2014)	Nested Case-control	USA	2002–2005	7.6	1905 M&F	45–84	Stroke/CHD	44/68
Ekim Seven(2015)	Nested Case-control	Denmark	1999–2006	11.4	6502 M&F	30–60	Stroke/CHD	179/297
Hamidreza Sabevr (2015)	Nested Case-control	USA	1990–1994	10	757 M&F	28–62	Stroke	119
Swapnil N.Rajpathak(2011)	Nested Case-control	USA	1993–1998	14	1944 F	50–79	Stroke	972
Jiankang Liu(2010)	Nested Case-control	USA	2000–2004	4	4571 M&F	21–94	Stroke/CHD	225/361
Naveed Sattar(2009)	Nested Case-control	British	1980–1996	16	1734 M	40–59	CHD	550
S. Söderberg(2004)	Nested Case-control	Sweden	1985–1994	4.9	828 M&F	25–74	Stroke	276
S. Söderberg (1999)	Nested Case-control	Sweden	1985–1994	9	190 M	25–74	CHD	62
Christof Prugger(2012)	Nested Case-control	Northern Ireland and France	1991–1994	10	240 M	50–59	Stroke	80
A. Michael Wallace(2001)	Nested Case-control	Scotland	1989–1991	5	1160 M	45–64	CHD	377
Debbie A Lawlor (2007)	Nested Case-control	UK	1999–2001	4	500 F	60–79	CHD	165
Jose (2005)	Case-control	India	—	—	140 F&M	52	CHD	94
JustoSierra-Johnson (2007)	Case-control	USA	1988–1994	—	6239 M&F	20–89	Stroke/CHD	160/228

M: male; F: female; CHD: coronary heart disease; yrs: years.

### Correlation analyses between leptin and CHD

**Fig 2:**
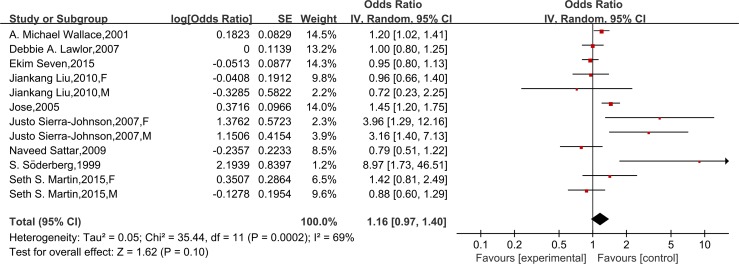
Forest plots of risk difference between leptin levels with CHD, adjusted for cardiovascular risk factors. Association of leptin and risk of CHD, adjusted for cardiovascular risk factors*. *All studies were adjusted for a minimum of smoking status, lipids, systolic blood pressure, and body mass index, except the studies of Liu JK, which did not adjust for lipids.

**Fig 3:**
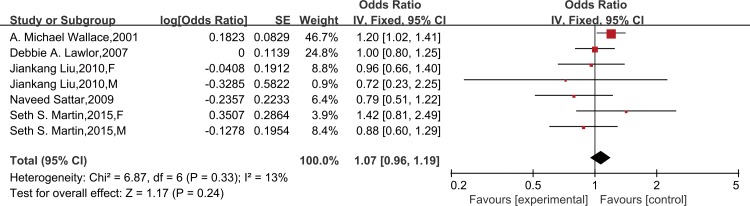
Forest plots of risk difference between leptin levels with CHD, adjusted for cardiovascular risk factors in high methodological quality studies. Association of leptin and risk of CHD in high methodological quality studies, adjusted for cardiovascular risk factors*. *All studies were adjusted for a minimum of smoking status, lipids, systolic blood pressure, and body mass index, except the studies of Liu JK, which did not adjust for lipids.

**Fig 4:**
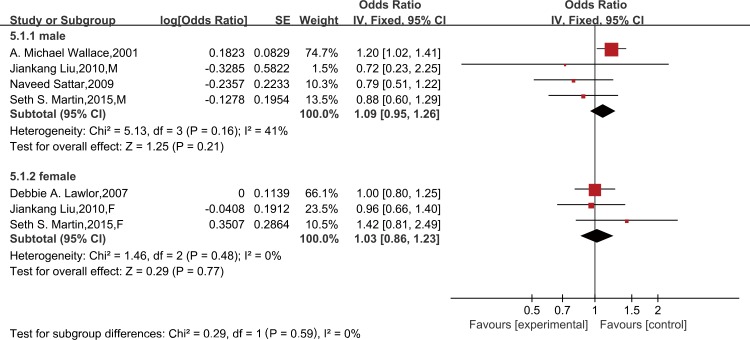
Gender difference between leptin levels and CHD, adjusted for cardiovascular risk factors. Gender difference of the association between leptin and risk of CHD, adjusted for cardiovascular risk factors*. *All studies were adjusted for a minimum of smoking status, lipids, systolic blood pressure, and body mass index, except the studies of Liu JK, which did not adjust for lipids.

Seven studies provided sociodemographics-adjusted data on the risk of CHD from leptin. Meta-analysis with a random-effect model indicated that leptin could significantly increase the risk of CHD (OR = 1.16, 95% CI 1.02–1.32; *P* for heterogeneity = 0.06, I^2^ = 46%) (S1 Fig). However, we found no association after further adjustment for other established cardiovascular risk factors in nine studies (OR = 1.16,95% CI = 0.97–1.40), and a random-effect model was applied for its homogeneous outcome (*P* for heterogeneity = 0.0002, I^2^ = 69%)(Fig 2). A further analysis was performed by excluding the two case-control studies, and this did not change the results (OR = 1.03, 95% CI 0.88–1.19; *P* for heterogeneity = 0.06, I^2^ = 46%)(S2 Fig). Moreover, analysis restricted to cardiovascular risk factors-adjusted data of high methodological quality (≥7 on the Newcastle-Ottawa Scale) was consistent with the findings (OR = 1.07, 95% CI 0.96–1.19; *P* for heterogeneity = 0.33, I^2^ = 13%) (Fig 3). In a subgroup meta-analysis, a high leptin level was not independently associated with CHD in females (OR = 1.03, 95% CI 0.86–1.23) or males (OR = 1.09, 95% CI 0.95–1.26) (Fig 4).

### Correlation analyses between leptin and stroke

**Fig 5:**
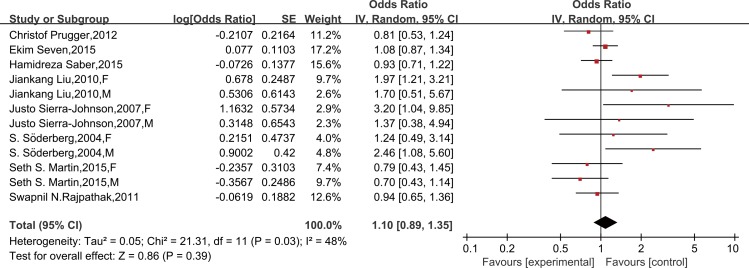
Forest plots of risk difference between leptin levels and stroke, adjusted for cardiovascular risk factors. Association of leptin and risk of stroke, adjusted for cardiovascular risk factors*. *All studies were adjusted for a minimum of smoking status, lipids, systolic blood pressure, and body mass index, except the studies of Liu JK, which did not adjust for lipids.

**Fig 6:**
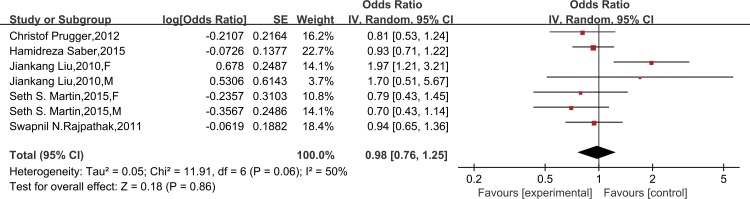
Forest plots of risk difference between leptin levels and stroke, adjusted for cardiovascular risk factors in high methodological quality studies. Association of leptin and risk of stroke in high methodological quality studies, adjusted for cardiovascular risk factors*. *All studies were adjusted for a minimum of smoking status, lipids, systolic blood pressure, and body mass index, except the studies of Liu JK, which did not adjust for lipids.

**Fig 7:**
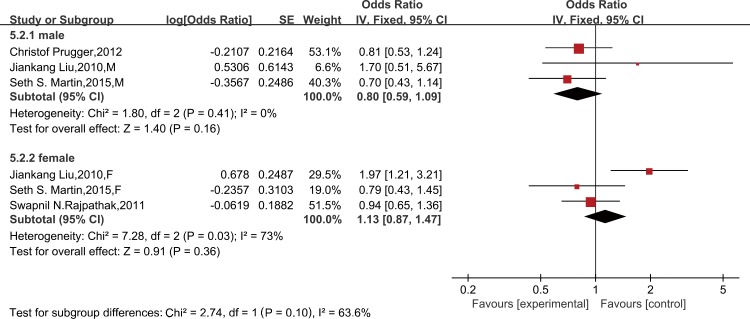
Gender difference between leptin levels and stroke, adjusted for cardiovascular risk factors. Gender difference of the association between leptin and risk of stroke, adjusted for cardiovascular risk factors*. *All studies were adjusted for a minimum of smoking status, lipids, systolic blood pressure, and body mass index, except the studies of Liu JK, which did not adjust for lipids.

Six studies provided sociodemographic-adjusted data on the risk of stroke and leptin. Meta-analysis using a random-effect model indicated that the risk of stroke did not increase in patients with high leptin (OR = 1.21, 95% CI 0.98–1.48; *P* for heterogeneity = 0.006, I^2^ = 63%) (S3 Fig). Similarly, the results remained the same after further adjustment for other established cardiovascular risk factors (OR = 1.10, 95% CI 0.89–1.35; *P* for heterogeneity = 0.03, I^2^ = 48%) (Fig 5) and after excluding the case-control study (OR = 1.03, 95% CI 0.91–1.17; *P* for heterogeneity = 0.04, I^2^ = 48%) (S4 Fig). A further analysis was restricted to cardiovascular risk factors-adjusted data of high methodological quality (≥7 on the Newcastle-Ottawa Scale) and is consistent with the findings (OR = 0.98, 95% CI 0.76–1.25; *P* for heterogeneity = 0.06, I^2^ = 50%) (Fig 6). In a subgroup meta-analysis, a high leptin level was not independently associated with stroke in females (OR = 1.13, 95% CI 0.87–1.47) or males (OR = 0.80, 95% CI 0.59–1.09) (Fig 7).

### Publication bias

Egger’s regression model was applied to test publication bias and did not indicate significant publication bias for CHD (*t =* 0.90, *P =* 0.391) or stroke (*t =* 1.42, *P =* 0.185).

## Discussion

This study reviewed and analyzed the results of thirteen studies investigating the effect of high leptin on the risks of developing CHD and stroke. Sociodemographic-adjusted studies indicated that high leptin was associated with an increased risk of CHD instead of stroke. After adjusting other established cardiovascular risk factors, they were not statistically significant both for CHD and stroke. Moreover, the result was consistent with cardiovascular risk factors-adjusted studies of high methodological quality.

A causal relation between leptin and CVD is not clear, although high leptin levels have been shown to be correlated with enhancing platelet aggregation and arterial thrombosis [16] and promoting angiogenesis [38]. Furthermore, leptin was reported to modulate inflammatory responses [39, 40] and induce proliferation and migration of vascular smooth muscle cells [41, 42]. However, the results of the research studying the association of leptin with CHD and stroke are inconsistent. In a recent prospective nested case-control study among 7051 population-based males and females within the Multi-Ethnic Study of Atherosclerosis (MESA), leptin levels were not associated with an increased 7.6-year risk of CVD events [26]. Similarly, results from another prospective nested case-control study suggested no association between serum leptin levels and risk of CVD among 6502 population-based participants within the Inter 99 study cohort during a mean follow-up time of 11.4 years [24]. However, in a 4-year follow-up of the Jackson Heart Study with 5170 participants, there is a significant association between leptin and stroke in women. One previous study including 1160 men showed leptin may be a novel, independent risk factor for CHD [35].

In our meta-analysis, a high baseline leptin level was not prospectively associated with CVD incidence in multivariable adjusted models, which was consistent with previous meta-analysis. Naveed Sattar et al. also failed to demonstrate a statistically significant association between leptin and CHD [32]. Moreover, in a meta-analysis of prior studies between leptin and stroke, the combined risk ratio across all studies was 1.09 (95% CI, 0.87–1.37) in the adjusted analyses [33]. It is reported that leptin levels increase with obesity and correlate significantly with body fat percentage and that the association with CVD may be partly influenced by genetic factors [43]. We conducted a further subgroup meta-analysis according to gender classification and the results remain as not statistically significant. Therefore, we inferred that leptin, as an acute response to stress or CVD, may not be causally linked to the risk of CVD and may just reflect a state of hypothalamic leptin resistance in obesity and one of the co-occurrences of multiple vascular risk factors with obesity.

There are certain limitations of our study. First, there was some heterogeneity among the studies in terms of sample size, duration of observation, number of events, and difference in criteria of a high leptin level. This heterogeneity may lead to a reduced statistical power for detecting a possible association between leptin level and CVD. Second, the adjustment for BMI in our analysis may potentially be “over-adjustment”. However, there were limited studies adjusting for all the common risk factors of CVD except for BMI. In addition, we evaluate leptin as a risk factor for CVD independent of obesity while accounting for other obesity effects. Third, we should be cautious interpreting the results because our studies were based on case-control studies and nested case-control studies, and these cannot yield causal relationships. Additional high-quality prospective cohort studies are needed to produce more reliable conclusions between the association of leptin and risk of CVD.

## Conclusion

In summary, the results of our meta-analysis indicated that leptin may not be associated with the risk of CVD. Studies based on larger well-designed prospective populations are still needed to clarify the causal relationship.

## Supporting information

S1 Checklist PRISMA Checklist. (DOC)

S1 Data Raw data of the present study. (RM5)

S1 File List of excluded full-text articles and reasons for exclusion. (DOCX)

S1 Fig Forest plots of risk difference between leptin levels and CHD, adjusted for sociodemographics. Association of leptin and risk of CHD, adjusted for cardiovascular sociodemographics *. *All studies were adjusted for age, race and town.
(TIF)

S2 Fig Forest plots of risk difference between leptin levels and CHD, adjusted for cardiovascular risk factors in nested case-control studies. Association of leptin and risk of CHD in nested case-control studies, adjusted for cardiovascular risk factors*. *All studies were adjusted for a minimum of smoking status, lipids, systolic blood pressure, and body mass index, except the studies of Liu JK, which did not adjust for lipids.
(TIF)

S3 Fig Forest plots of risk difference between leptin levels and stroke, adjusted for sociodemographics. Association of leptin and risk of stroke, adjusted for cardiovascular sociodemographics *. *All studies were adjusted for age, race and town.
(TIF)

S4 Fig Forest plots of risk difference between leptin levels and stroke, adjusted for cardiovascular risk factors in nested case-control studies. Association of leptin and risk of stroke in nested case-control studies, adjusted for cardiovascular risk factors*. *All studies were adjusted for a minimum of smoking status, lipids, systolic blood pressure, and body mass index, except the studies of Liu JK, which did not adjust for lipids.
(TIF)
