# Supplementary figures and images for: Leptin concentration and risk of coronary heart disease and stroke: A systematic review and meta-analysis
Source: PLoS One. 2017 Mar 9;12(3):e0166360. doi: 10.1371/journal.pone.0166360 (PMC5344319; doi:10.1371/journal.pone.0166360)

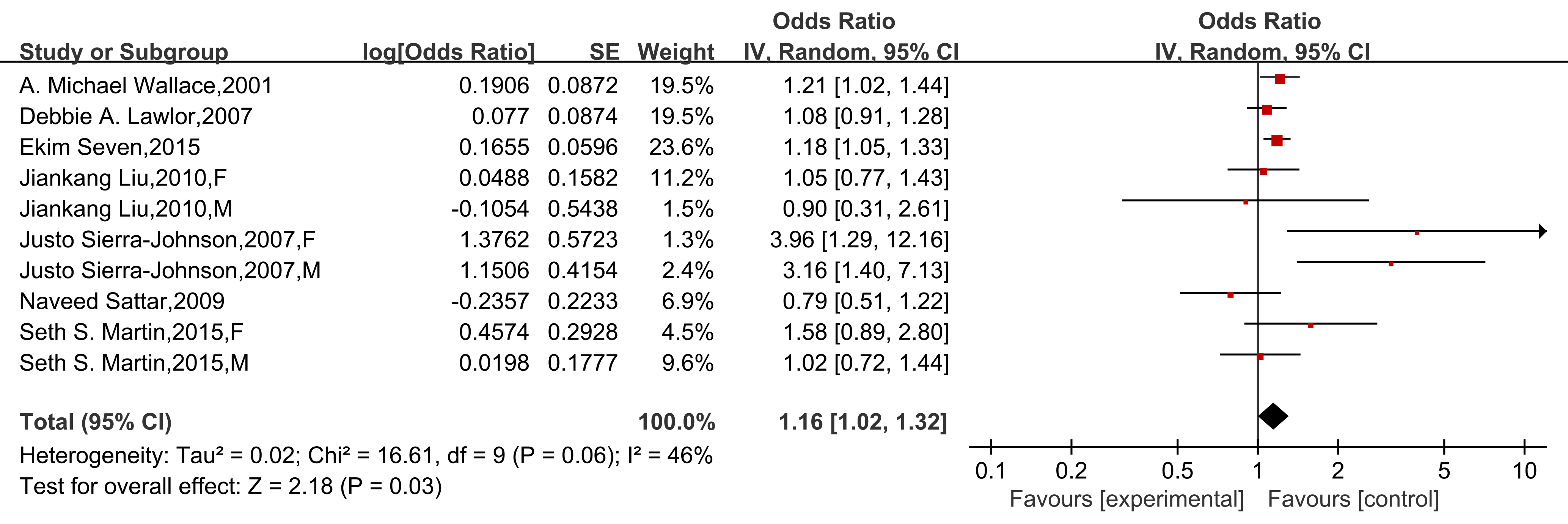

Supplement: S1 Fig — Association of leptin and risk of CHD, adjusted for cardiovascular sociodemographics *. *All studies were adjusted for age, race and town. (TIF) [file pone.0166360.s004.tif]

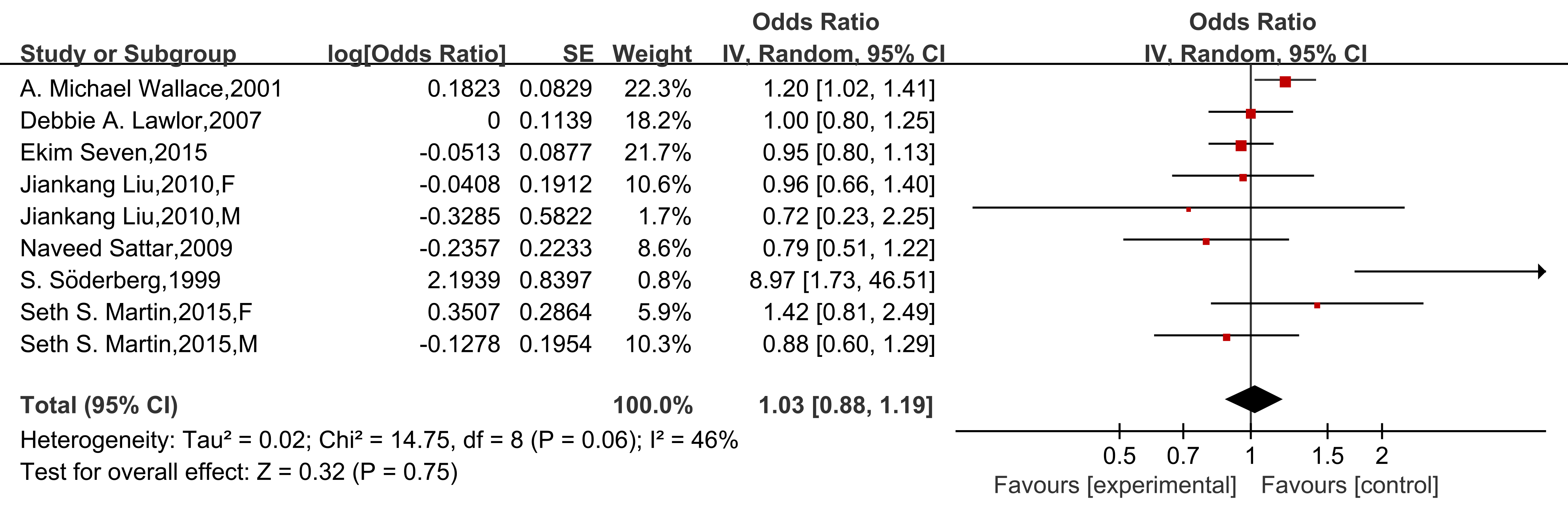

Supplement: S2 Fig — Association of leptin and risk of CHD in nested case-control studies, adjusted for cardiovascular risk factors*. *All studies were adjusted for a minimum of smoking status, lipids, systolic blood pressure, and body mass index, except the studies of Liu JK, which did not adjust for lipids. (TIF) [file pone.0166360.s005.tif]

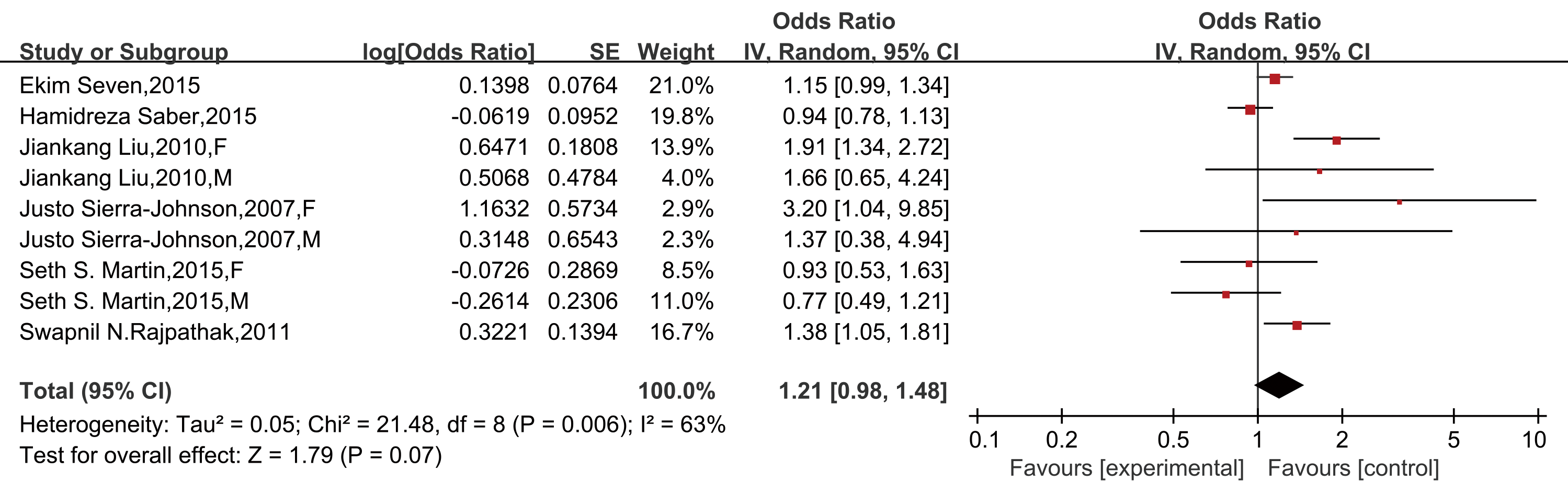

Supplement: S3 Fig — Association of leptin and risk of stroke, adjusted for cardiovascular sociodemographics *. *All studies were adjusted for age, race and town. (TIF) [file pone.0166360.s006.tif]

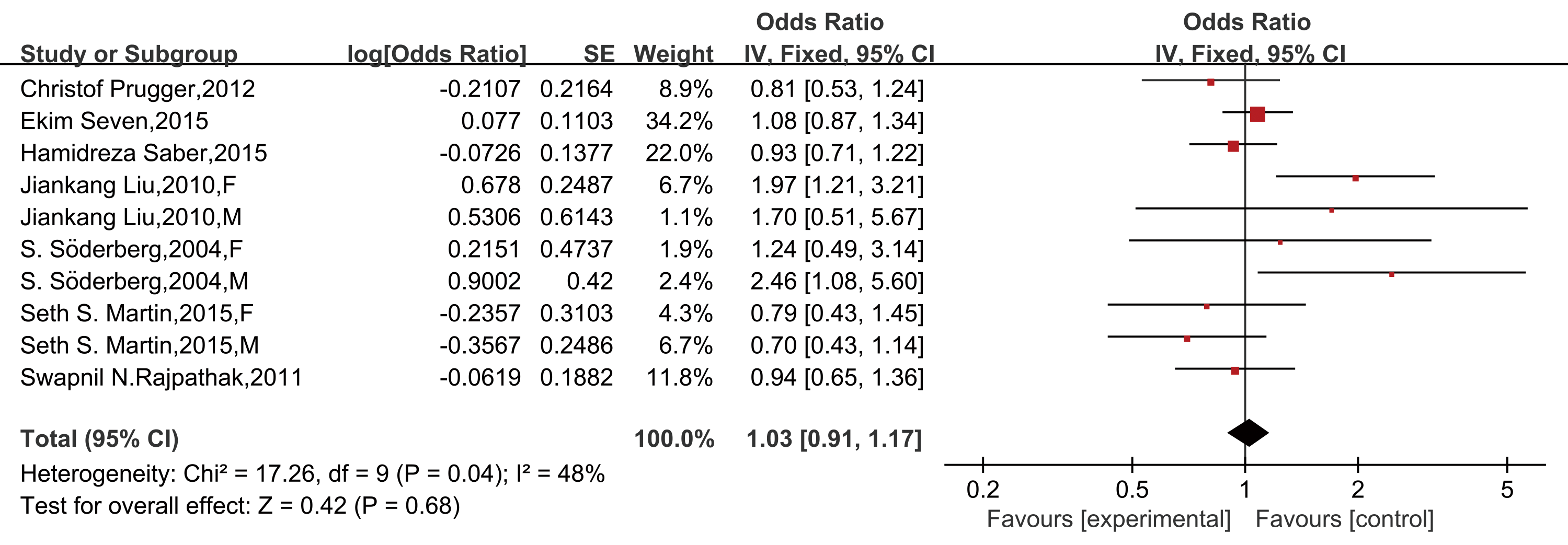

Supplement: S4 Fig — Association of leptin and risk of stroke in nested case-control studies, adjusted for cardiovascular risk factors*. *All studies were adjusted for a minimum of smoking status, lipids, systolic blood pressure, and body mass index, except the studies of Liu JK, which did not adjust for lipids. (TIF) [file pone.0166360.s007.tif]
