# Supplementary material for: Leptin concentration and risk of coronary heart disease and stroke: A systematic review and meta-analysis
Source: PLoS One. 2017 Mar 9;12(3):e0166360. doi: 10.1371/journal.pone.0166360 (PMC5344319; doi:10.1371/journal.pone.0166360)
Supplement: S1 File — (DOCX) [file pone.0166360.s003.docx]

Dear Editor，

A total of 4657 relevant studies were identified from the initial literature search, 90 of which appeared to be relevant to the meta-analysis following the subsequent selection. After careful screening and independent selection, 77 articles were excluded for various reasons, including base-line were CHD/stroke patients (n=8), articles not about leptin & CHD/stroke (n=41), without OR & 95% (n=10), reviews (n=18).Following was the detailed list of excluded articles:

**41 articles were not about leptin & stroke/CHD:**

1.	Uslu S, Kebapçı N, Kara M, Bal C. Relationship between adipocytokines and cardiovascular risk factors in patients with type 2 diabetes mellitus. Experimental and therapeutic medicine. 2012;4(1):113-20.

2.	Roszkowska-Gancarz M, Kurylowicz A, Polosak J, Mossakowska M, Franek E, Puzianowska-Kuźnicka M. Functional polymorphisms of the leptin and leptin receptor genes are associated with longevity and with the risk of myocardial infarction and of type 2 diabetes mellitus. Endokrynologia Polska. 2014;65(1):11-6.

3.	Adya R, Tan BK, Randeva HS. Differential effects of leptin and adiponectin in endothelial angiogenesis. Journal of diabetes research. 2015;2015.

4.	Charles LE, Burchfiel CM, Sarkisian K, Li S, Miller DB, Gu JK, et al. Leptin, adiponectin, and heart rate variability among police officers. American Journal of Human Biology. 2015;27(2):184-91.

5.	Kwaśniewska M, Kozińska J, Dziankowska-Zaborszczyk E, Kostka T, Jegier A, Rębowska E, et al. The impact of long-term changes in metabolic status on cardiovascular biomarkers and microvascular endothelial function in middle-aged men: a 25-year prospective study. Diabetology & metabolic syndrome. 2015;7(1):1.

6.	Nalini D, Karthick R, Shirin V, Manohar G, Malathi R. Role of the adipocyte hormone leptin in cardiovascular diseases–a study from Chennai based Population. Thrombosis journal. 2015;13(1):1.

7.	Reddy RKY, Mahendra J, Gurumurthy P. Identification of predictable biomarkers in conjunction to Framingham risk score to predict the risk for cardiovascular disease (CVD) in Non cardiac subjects. Journal of clinical and diagnostic research: JCDR. 2015;9(2):BC23.

8.	Rhee CM, Nguyen DV, Moradi H, Brunelli SM, Dukkipati R, Jing J, et al. Association of Adiponectin With Body Composition and Mortality in Hemodialysis Patients. American Journal of Kidney Diseases. 2015;66(2):313-21.

9.	Seven E. Overweight, hypertension and cardiovascular disease: focus on adipocytokines, insulin, weight changes and natriuretic peptides. Danish medical journal. 2015;62(11).

10.	Jaleel A, Aqil S, Jaleel S, Jaleel F. Adipocytokines in subjects with and without ischemic cerebrovascular disease. Acta Neurologica Belgica. 2010;110(3):234.

11.	Khalili P, Flyvbjerg A, Frystyk J, Lundin F, Jendle J, Engström G, et al. Total adiponectin does not predict cardiovascular events in middle-aged men in a prospective, long-term follow-up study. Diabetes & metabolism. 2010;36(2):137-43.

12.	Kaur S, Zilmer K, Leping V, Zilmer M. The levels of adiponectin and leptin and their relation to other markers of cardiovascular risk in patients with psoriasis. Journal of the European Academy of Dermatology and Venereology. 2011;25(11):1328-33.

13.	Andreasson AN, Undén AL, Elofsson S, Brismar K. Leptin and adiponectin: distribution and associations with cardiovascular risk factors in men and women of the general population. American Journal of Human Biology. 2012;24(5):595-601.

14.	King GA, Deemer SE, Thompson DL. Adiponectin is associated with risk of the metabolic syndrome and insulin resistance in women. Acta diabetologica. 2012;49(1):41-9.

15.	Messaaoui A, Willems D, Mélot C, Dorchy H. Risk markers for cardiovascular disease in young type 1 diabetic patients: lipoproteins, high-sensitivity C-reactive protein and adiponectin. Acta Clinica Belgica. 2012;67(2):79-82.

16.	Saito I, Yamagishi K, Chei C-L, Cui R, Ohira T, Kitamura A, et al. Total and high molecular weight adiponectin levels and risk of cardiovascular disease in individuals with high blood glucose levels. Atherosclerosis. 2013;229(1):222-7.

17.	Sarvottam K, Magan D, Yadav RK, Mehta N, Mahapatra SC. Adiponectin, interleukin-6, and cardiovascular disease risk factors are modified by a short-term yoga-based lifestyle intervention in overweight and obese men. The Journal of Alternative and Complementary Medicine. 2013;19(5):397-402.

18.	Ketlogetswe KS, Post WS, Xiuhong L, Palella Jr F, Jacobson LP, Margolick JB, et al. Lower adiponectin is associated with subclinical cardiovascular disease among HIV-infected men. AIDS (London, England). 2014;28(6):901.

19.	Singhal A, Farooqi IS, Cole TJ, O’Rahilly S, Fewtrell M, Kattenhorn M, et al. Influence of leptin on arterial distensibility a novel link between obesity and cardiovascular disease? Circulation. 2002;106(15):1919-24.

20.	Choi K, Lee J, Lee K, Seo J, Oh J, Kim S, et al. Serum adiponectin concentrations predict the developments of type 2 diabetes and the metabolic syndrome in elderly Koreans. Clinical endocrinology. 2004;61(1):75-80.

21.	Qi L, Doria A, Manson JE, Meigs JB, Hunter D, Mantzoros CS, et al. Adiponectin genetic variability, plasma adiponectin, and cardiovascular risk in patients with type 2 diabetes. Diabetes. 2006;55(5):1512-6.

22.	Kuller LH, Grandits G, Cohen JD, Neaton JD, Prineas R, Group MRFITR. Lipoprotein particles, insulin, adiponectin, C-reactive protein and risk of coronary heart disease among men with metabolic syndrome. Atherosclerosis. 2007;195(1):122-8.

23.	Lim S, Koo BK, Cho SW, Kihara S, Funahashi T, Cho YM, et al. Association of adiponectin and resistin with cardiovascular events in Korean patients with type 2 diabetes: the Korean atherosclerosis study (KAS): a 42-month prospective study. Atherosclerosis. 2008;196(1):398-404.

24.	Magno CP, Araneta MRG, Macera CA, Anderson GW. Cardiovascular disease prevalence, associated risk factors, and plasma adiponectin levels among Filipino American women. women. 2008;5:7.

25.	Kaisar MO, Armstrong K, Hawley C, Campbell S, Mudge D, Johnson DW, et al. Adiponectin is associated with cardiovascular disease in male renal transplant recipients: baseline results from the LANDMARK 2 study. BMC nephrology. 2009;10(1):1.

26.	Bao B, Xu L, Jiang C, Jin Y, Liu B, Thomas G, et al. [Relation of serum leptin and adiponectin to cardiovascular risk factors in older adults: a Guangzhou biobank cohort study-CVD]. Zhonghua liu xing bing xue za zhi= Zhonghua liuxingbingxue zazhi. 2010;31(2):121-5.

27.	Hajmohammadi T, Sadeghi M, Dashti M, Hashemi M, Saadatnia M, Soghrati M, et al. Relationship between carotid intima-media thickness with some inflammatory biomarkers, ghrelin and adiponectin in Iranians with and without metabolic syndrome in Isfahan cohort study. ARYA Atheroscler. 2010;6(2).

28.	Yamashita T, Sasahara T, Pomeroy SE, Collier G, Nestel PJ. Arterial compliance, blood pressure, plasma leptin, and plasma lipids in women are improved with weight reduction equally with a meat-based diet and a plant-based diet. Metabolism: clinical and experimental. 1998;47(11):1308-14.

29.	Al‐Daghri N, Al‐Rubean K, Bartlett W, Al‐Attas O, Jones A, Kumar S. Serum leptin is elevated in Saudi Arabian patients with metabolic syndrome and coronary artery disease. Diabetic medicine. 2003;20(10):832-7.

30.	Abdella N, Mojiminiyi O, Moussa M, Zaki M, Al Mohammedi H, Al Ozairi E, et al. Plasma leptin concentration in patients with Type 2 diabetes: relationship to cardiovascular disease risk factors and insulin resistance. Diabetic medicine. 2005;22(3):278-85.

31.	Mayer O, Filipovský J, Galovcová M, Bruthans J, Hrbkova J, Vrzalova J, et al. An inverse association between serum leptin concentration and reported alcohol intake in patients with manifest vascular disease. European journal of clinical nutrition. 2010;64(11):1350-7.

32.	Sinnaeve P, Moreels S, Vydt T, Dubois C, Adriaenssens T, Coosemans M, et al., editors. Leptin-Adiponectin ratio does not predict outcome after ACS. European Heart Journal Supplements; 2010: Saunders.

33.	Rathmann W, Haastert B, Herder C, Hauner H, Koenig W, Meisinger C, et al. Differential association of adiponectin with cardiovascular risk markers in men and women? The KORA survey 2000. International journal of obesity. 2007;31(5):770-6.

34.	Shioji K, Moriwaki S, Takeuchi Y, Uegaito T, Mutsuo S, Matsuda M. Relationship of serum adiponectin level to adverse cardiovascular events in patients who undergo percutaneous coronary intervention. Circulation Journal. 2007;71(5):675-80.

35.	Wannamethee SG, Tchernova J, Whincup P, Lowe GD, Kelly A, Rumley A, et al. Plasma leptin: associations with metabolic, inflammatory and haemostatic risk factors for cardiovascular disease. Atherosclerosis. 2007;191(2):418-26.

36.	Dunbar JC, Lu H. Chronic intracerebroventricular insulin attenuates the leptin-mediated but not alpha melanocyte stimulating hormone increase in sympathetic and cardiovascular responses. Brain research bulletin. 2000;52(2):123-6.

37.	Matsuda M, Shimomura I, Sata M, Arita Y, Nishida M, Maeda N, et al. Role of adiponectin in preventing vascular stenosis The missing link of adipo-vascular axis. Journal of Biological Chemistry. 2002;277(40):37487-91.

38.	Ishikawa Y, Akasaka Y, Ishii T, Yoda‐Murakami M, Choi‐Miura NH, Tomita M, et al. Changes in the distribution pattern of gelatin‐binding protein of 28 kDa (adiponectin) in myocardial remodelling after ischaemic injury. Histopathology. 2003;42(1):43-52.

39.	Pellme F, Smith U, Funahashi T, Matsuzawa Y, Brekke H, Wiklund O, et al. Circulating adiponectin levels are reduced in nonobese but insulin-resistant first-degree relatives of type 2 diabetic patients. Diabetes. 2003;52(5):1182-6.

40.	Zietz B, Herfarth H, Paul G, Ehling A, Müller-Ladner U, Schölmerich J, et al. Adiponectin represents an independent cardiovascular risk factor predicting serum HDL-cholesterol levels in type 2 diabetes. FEBS letters. 2003;545(2):103-4.

41.	Couillard C, Mauriege P, Prud'Homme D, Nadeau A, Tremblay A, Bouchard C, et al. Plasma leptin concentrations: gender differences and associations with metabolic risk factors for cardiovascular disease. Diabetologia. 1997;40(10):1178-84.

**18 articles were reviews**

1.	Hou N, Luo JD. Leptin and cardiovascular diseases. Clinical and Experimental Pharmacology and Physiology. 2011;38(12):905-13.

2.	Hughes-Austin JM, Larsen BA, Allison MA. Visceral adipose tissue and cardiovascular disease risk. Current Cardiovascular Risk Reports. 2013;7(2):95-101.

3.	Savopoulos C, Michalakis K, Apostolopoulou M, Miras A, Hatzitolios A. Adipokines and stroke: a review of the literature. Maturitas. 2011;70(4):322-7.

4.	Raman K, Chong M, Akhtar-Danesh G-G, D'Mello M, Hasso R, Ross S, et al. Genetic markers of inflammation and their role in cardiovascular disease. Canadian Journal of Cardiology. 2013;29(1):67-74.

5.	Signore AP, Zhang F, Weng Z, Gao Y, Chen J. Leptin neuroprotection in the CNS: mechanisms and therapeutic potentials. Journal of neurochemistry. 2008;106(5):1977-90.

6.	Beltowski J. Leptin and atherosclerosis. Atherosclerosis. 2006;189(1):47-60.

7.	Aronne LJ, Brown WV, Isoldi KK. Cardiovascular disease in obesity: A review of related risk factors and risk-reduction strategies. Journal of clinical lipidology. 2007;1(6):575-82.

8.	Goldstein BJ, Scalia R. Adipokines and vascular disease in diabetes. Current diabetes reports. 2007;7(1):25-33.

9.	Hopkins TA, Ouchi N, Shibata R, Walsh K. Adiponectin actions in the cardiovascular system. Cardiovascular research. 2007;74(1):11-8.

10.	Kaisar OM, Johnson DW, Prins JB, Isbel N. The role of novel biomarkers of cardiovascular disease in chronic kidney disease: focus on adiponectin and leptin. Current cardiology reviews. 2008;4(4):287-92.

11.	Marinou K, Tousoulis D, Antonopoulos AS, Stefanadi E, Stefanadis C. Obesity and cardiovascular disease: from pathophysiology to risk stratification. International journal of cardiology. 2010;138(1):3-8.

12.	Sarvottam K, Yadav RK. Obesity-related inflammation & cardiovascular disease: Efficacy of a yoga-based lifestyle intervention. The Indian journal of medical research. 2014;139(6):822.

13.	Mark AL, Sivitz WI. Uncoupling metabolism and coupling leptin to cardiovascular disease. Arteriosclerosis, thrombosis, and vascular biology. 2002;22(6):881-3.

14.	Korolczuk A, Dudka J. Increased risk of cardiovascular complications in chronic kidney disease: a possible role of leptin. Current pharmaceutical design. 2014;20(4):666-74.

15.	Luo J-D, Zhang G-S, Chen M-S. Leptin and cardiovascular diseases. Drug News Perspect. 2005;18(7):427.

16.	Mansoub S, Chan MK, Adeli K. Gap analysis of pediatric reference intervals for risk biomarkers of cardiovascular disease and the metabolic syndrome. Clinical biochemistry. 2006;39(6):569-87.

17.	Martin SS, Qasim A, Reilly MP. Leptin resistance: a possible interface of inflammation and metabolism in obesity-related cardiovascular disease. Journal of the American College of Cardiology. 2008;52(15):1201-10.

18.	Mattu HS, Randeva HS. Role of adipokines in cardiovascular disease. Journal of Endocrinology. 2013;216(1):T17-T36.

**10 articles without OR & 95%CI**

1.	Akram S, Ahmed Z, Fayyaz I, Mehmood S, Ghani M, Choudhary AM, et al. Serum leptin levels in patients with coronary artery disease. J Ayub Med Coll Abbottabad. 2011;23(4):13-4.

2.	Amrock SM, Weitzman M. Effect of increased leptin and C-reactive protein levels on mortality: Results from the National Health and Nutrition Examination Survey. Atherosclerosis. 2014;236(1):1-6.

3.	Atwa M, Emara A, Balata M, Youssef N, Bayoumy N, Sherif A, et al. Serum leptin, adiponectin, and resistin among adult patients with acanthosis nigricans: correlations with insulin resistance and risk factors for cardiovascular disease. International journal of dermatology. 2014;53(10):e410-e20.

4.	Batsis JA, Sahakyan KR, Singh P, Bartels SJ, Somers VK, Lopez-Jimenez F, editors. Leptin, Adiposity, and Mortality: Results From the National Health and Nutrition Examination Survey III, 1988 to 1994. Mayo Clinic Proceedings; 2015: Elsevier.

5.	Bian X, Liu N, Bai Y, Zheng L, He P, Su X, et al. Association of leptin with mortality in patients on maintenance hemodialysis: a prospective study. Iranian journal of kidney diseases. 2014;8(4):314.

6.	Li W-C, Hsiao K-Y, Chen I-C, Chang Y-C, Wang S-H, Wu K-H. Serum leptin is associated with cardiometabolic risk and predicts metabolic syndrome in Taiwanese adults. Cardiovasc Diabetol. 2011;10(36.10):1186.

7.	Pala L, Monami M, Ciani S, Dicembrini I, Pasqua A, Pezzatini A, et al. Adipokines as possible new predictors of cardiovascular diseases: a case control study. Journal of nutrition and metabolism. 2011;2012.

8.	Romero-Corral A, Sierra-Johnson J, Lopez-Jimenez F, Thomas RJ, Singh P, Hoffmann M, et al. Relationships between leptin and C-reactive protein with cardiovascular disease in the adult general population. Nature Clinical Practice Cardiovascular Medicine. 2008;5(7):418-25.

9.	Shahouzehi B, Shokoohi M, Najafipour H. The effect of opium addiction on serum adiponectin and leptin levels in male subjects: a case control study from kerman coronary artery disease risk factors study (KERCADRS). Excli Journal. 2013;12:916-23.

10.	Abu-Farha M, Behbehani K, Elkum N. Comprehensive analysis of circulating adipokines and hsCRP association with cardiovascular disease risk factors and metabolic syndrome in Arabs. Cardiovasc Diabetol. 2014;13(76):76-13.

**8 articles included stroke/CHD at baseline**

1.	Hall J, Vora N, Langworthy R, Stock S, Momin A, Sherwood R, et al. Leptin/adiponectin ratio in patients with coronary heart disease: comparing subjects with and without metabolic syndrome. Annals of clinical biochemistry. 2011;48(4):327-31.

2.	Kajikawa Y, Ikeda M, Takemoto S, Tomoda J, Ohmaru N, Kusachi S. Association of circulating levels of leptin and adiponectin with metabolic syndrome and coronary heart disease in patients with various coronary risk factors. International heart journal. 2011;52(1):17-22.

3.	Wallerstedt S, Eriksson AL, Niklason A, Ohlsson C, Hedner T. Serum leptin and myocardial infarction in hypertension. Blood pressure. 2004;13(4):243-6.

4.	Wannamethee SG, Shaper AG, Whincup PH, Lennon L, Sattar N. Obesity and risk of incident heart failure in older men with and without pre-existing coronary heart disease: does leptin have a role? Journal of the American College of Cardiology. 2011;58(18):1870-7.

5.	Welsh P, Murray HM, Buckley BM, De Craen AJ, Ford I, Jukema JW, et al. Leptin Predicts Diabetes but Not Cardiovascular Disease Results from a large prospective study in an elderly population. Diabetes care. 2009;32(2):308-10.

6.	Wolk R, Berger P, Lennon RJ, Brilakis ES, Johnson BD, Somers VK. Plasma leptin and prognosis in patients with established coronary atherosclerosis. Journal of the American College of Cardiology. 2004;44(9):1819-24.

7.	Zoccali C, Mallamaci F, Tripepi G, Benedetto FA, Cutrupi S, Parlongo S, et al. Adiponectin, metabolic risk factors, and cardiovascular events among patients with end-stage renal disease. Journal of the American Society of Nephrology. 2002;13(1):134-41.

8.	Carbone F, Burger F, Roversi G, Tamborino C, Casetta I, Seraceni S, et al. Leptin/adiponectin ratio predicts poststroke neurological outcome. European journal of clinical investigation. 2015;45(11):1184-91.
